# Mutation of the Sp1 binding site in the 5′ flanking region of *SRY* causes sex reversal in rabbits

**DOI:** 10.18632/oncotarget.16979

**Published:** 2017-04-09

**Authors:** Yuning Song, Tingjun Liu, Yong Wang, Jichao Deng, Mao Chen, Lin Yuan, Yi Lu, Yuxin Xu, Haobin Yao, Zhanjun Li, Liangxue Lai

**Affiliations:** ^1^ Jilin Provincial Key Laboratory of Animal Embryo Engineering, Jilin University, Changchun, China; ^2^ Key Laboratory of Regenerative Biology, and Guangdong Provincial Key Laboratory of Stem Cells and Regenerative Medicine, South China Institute for Stem Cell Biology and Regenerative Medicine, Guangzhou Institutes of Biomedicine and Health, Chinese Academy of Sciences, Guangzhou, Guangdong, China

**Keywords:** Sp1, SRY, CRISPR/Cas9, sex reversed, Pathology Section

## Abstract

Sex-determining region Y is a crucial gene that initiates male sex determination in mammals. Mutations of the Sp1-binding site in the 5′ flanking region of *SRY* are associated with clinical male-to-female sex reversal syndrome, although such occurrences are rare and, until now, have not been reported in animal models. In this study, we mutated Sp1-binding sites in the 5′ flanking region of the rabbit *SRY* gene using the CRISPR/Cas9 system. As expected, the *SRY*-Sp1 knockout rabbits had female external and internal genitalia and exhibited normal female copulatory behaviors, but they were infertile, and the adults displayed reduced follicles. Interestingly, we successfully obtained offspring from sex-reversed *SRY*-Sp1 knockout rabbits using embryo transfer. In summary, our study demonstrates that Sp1 is a major regulator in *SRY* gene transcription, and mutations of the Sp1 binding sites (Sp1-B and Sp1-C) in the 5′ flanking region of *SRY* induce sex reversal in rabbits, which can be used as targets for clinical research of male-to-female sex reversal syndrome. Additionally, we provide the first evidence that sex reversal syndrome patients have the potential to become pregnant with the use of embryo transfer.

## INTRODUCTION

In mammals, the sex-determining gene, *SRY*, which is expressed specifically in the genital ridges, facilitates gender determination [1, 2]. Mutation or dysfunction of the HMG box, a conserved DNA-binding domain of the *SRY* protein, have been shown to induce male-to-female sex reversal syndrome in XY individuals [3, 4]. In addition, mutations in the 5′ regulatory region of the *SRY* gene were also responsible for sex reversal in clinical studies [5]. A naturally occurring deletion in the 5′ region of *SRY* affecting the Sp1 binding site is known to be associated with sex reversal in humans, indicating that mutation of the Sp1 binding site in the 5′ flanking region of *SRY* causes sex reversal [6].

The Sp1 transcription factor binds to G-C rich motifs that are 21-22 bp long. It plays a role as a transcriptional activator in most mammalian genes and is essential for the differentiation of spermatids [7, 8]. Co-transfection experiments suggest that two Sp1 binding sites located in the 5′ flanking region are associated with transcriptional activation of *SRY* in humans [9]. Currently, there is a need to further characterize the association between mutations in Sp1 binding sites and female sex reversal and to provide proper animal models for clinical research of male-to-female sex reversal syndromes.

Here, we mutated Sp1-binding sites in the 5′ flanking region of rabbit *SRY* using CRISPR/Cas9, and observed the typical phenotype of sex reversal syndromes in *SRY*-Sp1 knockout (KO) rabbits. Additionally, we identified the potential ability of sex-reversed females as surrogates for embryo transfer in the rabbit model.

## RESULTS

### Conserved Sp1 binding sites in the 5′ flanking sequence of rabbit SRY

**Figure 1:**
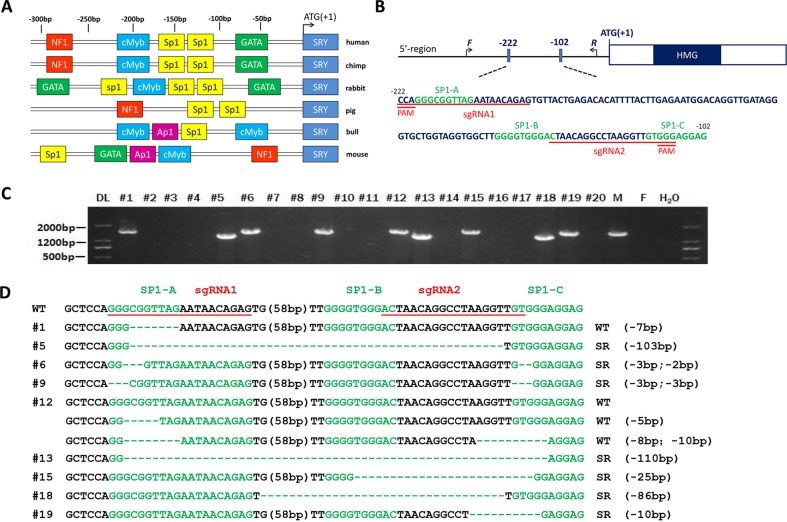
Generation of the SRY-Sp1 KO, XY rabbits using the Cas9/gRNA system **A**. Schematic representation of the positions corresponding to the different potential regulatory elements identified in the 5′ flanking region of the *SRY* gene of different species. Identified putative binding sites are indicated by different colored boxes on the sequence, indicating a position in the sense or antisense strand. **B**. Schematic diagram of the 2 sgRNA target sites in the Sp1 binding site located upstream of the *SRY* locus. The Sp1 binding sites are indicated in green. sgRNA target sites are underlined and highlighted in red. F and R represent the PCR primer pairs used for mutation detection. **C**. Mutation determination of the *SRY*-Sp1 KO, XY newborn rabbits by PCR. Primers used were *SRY*-F and R (Supplementary Table S1). DL, DNA marker III. F: wild type female rabbit; M: wild type male rabbit. **D**. T-cloning sequences of mutant alleles in SRY-Sp1 KO newborn rabbits. The target sequences are underlined in red; the Sp1 binding sites are green; deletions (−) and insertions (+) are shown. WT, wild type control.

To identify the conserved Sp1 binding sites in different species, the 5′ flanking region sequences of human, chimpanzee, rabbit, mouse, bull and pig *SRY* genes were comparatively analyzed using BLAST. Results depicted in Figure 1A illustrate that human and rabbit Sp1 binding sites shared maximum homology as identified by TF searcher, indicating that rabbits are the most suitable animal model to investigate the role of Sp1 in male-to-female sex reversal syndrome.

To disrupt the Sp1-binding sites located in the 5′ flanking region of rabbit *SRY*, we initially designed two guide RNAs (gRNAs) to target the Sp1-A and Sp1-C binding sites (Figure 1B). Then, we tested the efficacy of the RNA-guided Cas9 nucleases in cells and zygotes. As shown in Supplementary Figure S1, mutations in the Sp1 binding site can be achieved *via* the CRISPR/Cas 9 system with high efficiency in cells and zygotes.

### Generation of *SRY*-Sp1 KO rabbits using the CRISPR/CAS9 system

To generate *SRY*-Sp1 KO rabbits, we transferred 391 embryos to eight pseudo-pregnant recipient rabbits. Five recipients carried the pregnancies to term and gave birth to 20 live pups (Table 1). Genomic DNA samples from F0-generation pups were extracted and tested for Sp1 mutations. Nine of the F0 pups were male and after T-cloning and PCR-sequencing, each of them was identified as carrying the KO allele (Figure 1C). Sp1 binding site mutations were found in all modified pups (Figure 1D). We detected a large fragment deletion spanning three Sp1 binding sites in pup #13, while pups #5, #6, #9, #12, #15 and #18 carried two Sp1 binding site mutations each (Supplementary Table S2).

The potential off-target (POT) effects in these genetically modified rabbits were determined by Sanger sequencing and T7E1. The results revealed that no off-target effects occurred in the Sp1 KO, XY rabbits (Supplementary Figure S2).

**Table 1:** Generation of genetically targeted SRY-sp1-KO rabbits using CRISPR/Cas9 system

Recipients	gRNA/Cas9 mRNA(ng/uL)	Embryos injected	Embryos transferred (%microinjected)	Pregnancy	Pups obtained (%transferred)	Pregnancy (%pups)	Pups obtained (%male pups)	Pups with Sex reversal
1	25/100	50	42(84%)	NO				
2	25/100	45	40(89%)	YES	6(15%)	2(33.3%)	2(100%)	2
3	25/100	48	40(83.3%)	NO				
4	25/100	45	42(93.3%)	YES	4(9.52%)	2(50%)	2(100%)	1
5	25/100	53	48(93%)	YES	3(6.25%)	2(66.7%)	2(100%)	1
6	25/100	48	45(93.75%)	NO				
7	25/100	52	50(96.1%)	YES	3(6%)	1(33.3%)	1(100%)	1
8	25/100	50	48(96%)	YES	4(8.3%)	2(50%)	2(100%)	2

### Mutation of Sp1-B and Sp1-C causes male-to-female sex reversal in rabbits

**Figure 2:**
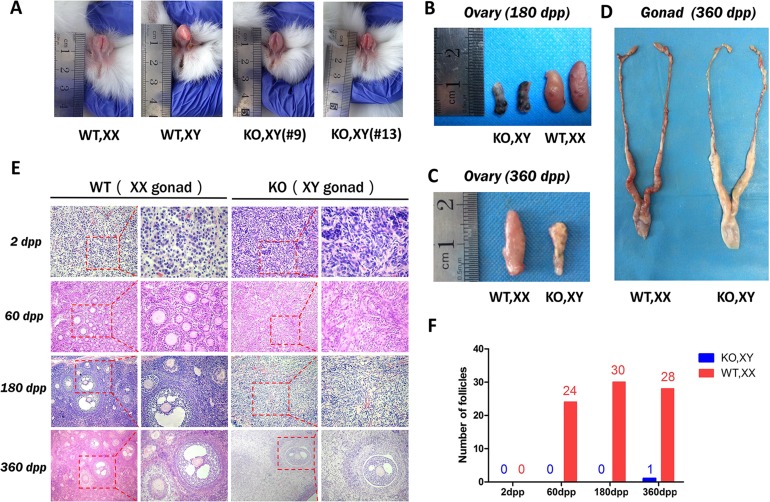
Sex reversal in SRY-Sp1 KO, XY rabbits **A**. External genitalia of the KO, XY rabbits and wild type rabbits. WT, XX: wild type female rabbit; WT, XY: wild type male rabbit; KO, XY: the *SRY*-Sp1 KO rabbit. **B**. The ovaries of the KO, XY rabbit (#13) and the WT, XX rabbit at 180 days. WT, wild type control. **C**. The ovaries of the KO, XY rabbit (#18) and the WT, XX rabbit at 360 days. **D**. The reproductive organs of the KO, XY rabbit (#18) and the WT, XX rabbit at 360 days. **E**. H&E staining of the ovaries from WT, XX and KO, XY rabbits at different ages. WT, XX, wild type female control. **F**. Statistical analysis of the follicle numbers in the KO, XY and WT, XX rabbits. WT, XX: wild type female rabbit.

To determine whether the mutations in the Sp1 binding sites induced sex reversal, we examined the external genitalia of the Sp1 KO, XY rabbits when the pups were two months old. As shown in Figure 2A, 7 of 9 Sp1 KO, XY rabbits had completely female external genitalia, while the other two (#1 and #12) had male external genitalia. More importantly, we found that disruption of only the Sp1-B (#18) or the Sp1-C (#19) binding sites can cause sex reversal, but disruption of only the Sp1-A site (#1) has no effect on sex development (Figure 1D and Supplementary Table S2), demonstrating that the Sp1-B and Sp1-C binding sites are necessary for transcriptional regulation of *SRY*. This result is consistent with previous studies using human cells [9].

Next, we examined the internal genitalia of the sex-reversed Sp1 KO, XY pups. These rabbits had significantly smaller ovaries compared with those of the WT, XX rabbits (Figure 2B and 2C). Few follicles were observed by H & E staining in the KO, XY ovaries at 60, 180 and 360 days post-partum (*dpp*), consistent with developmental disorders of ovarian interstitial cells (Figure 2E). The reduction in the number of follicles was also statistically significant (Figure 2F), indicating reduced follicles and ovulatory dysfunction in the KO, XY ovaries.

Interestingly, similar and normal genital systems were observed in the KO, XY and WT, XX gonads (Figure 2D), H & E staining results confirmed that there were no significant differences between the KO, XY and WT, XX rabbits in the oviduct (Supplementary Figure S3), uterus (Supplementary Figure S4) or cervix (Supplementary Figure S5).

These results suggest that mutations of the Sp1-B or Sp1-C site cause male-to-female sex reversal in rabbits with complete external and internal female genitalia. However, ovulation disorders were determined in the SRY-Sp1 KO rabbits.

### Reduced gene expression of *SRY* in SRY-Sp1 rabbits

**Figure 3:**
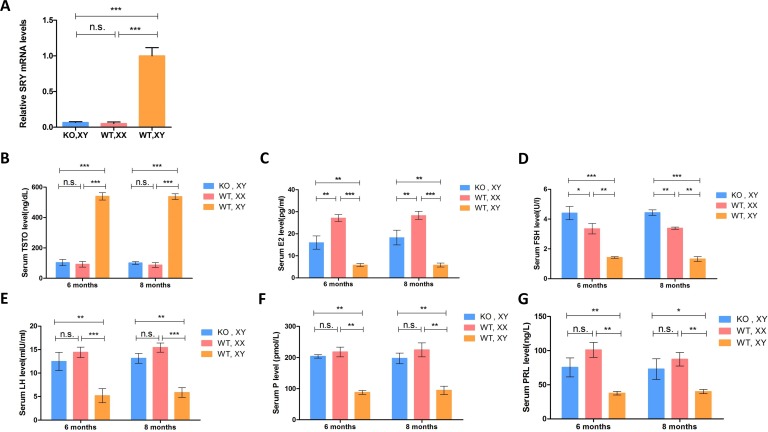
Expression of the SRY gene and sex hormone determination in SRY-Sp1 KO, XY rabbits **A**. Expression of the *SRY* gene was determined by qRT-PCR. A probability of *p* < 0.05 was considered statistically significant. *, *p* < 0.05; **, *p* < 0.01; ***, *p* < 0.005; WT, XX, wild type female rabbit; WT, XY, wild type male rabbit; KO, XY, *SRY*-Sp1 KO rabbit. **B**.-**G**. Sex hormones were determined using ELISA. The results are presented as the mean±SEM. Data were analyzed with one-way ANOVA using GraphPad Prism software.

To explore the mechanisms of sex reversal and the effect of Sp1 transcription factor on the expression of the *SRY* gene in the KO, XY rabbits, we assayed *SRY* gene expression using qRT-PCR in the KO, XY gonads at 15 days post coitum (*dpc)*, which was determined in previous studies to be the time of highest *SRY* expression [10]. The results showed that *SRY* expression in KO, XY gonads was dramatically reduced compared to the WT, XY gonads, but was not significantly different from expression in the WT, XX gonads (Figure 3A). This observation demonstrates that the Sp1 binding sites, which are adjacent to the initiation codon and are highly similar to those of humans, may facilitate transcription of the *SRY* gene. The development of female-type gonads in XY rabbits that lack *SRY* function lends support to the notion that the key role for *SRY* is both activation of the testis-determining pathway and suppression of the ovarian-determining pathway.

### Fertility of the *SRY*-Sp1 KO, XY rabbits

To test the fertility of the *SRY-*Sp1 KO, XY rabbits, we mated them with WT males in which fertility was previously confirmed. Unfortunately, we did not detect any pregnancies in the KO, XY rabbits, even though their copulatory behavior was similar to that of normal females. Even when the rabbits were injected with ovulation stimulating hormones (follicle-stimulating hormone and luteinizing hormone) before mating, pregnancy was not observed. We concluded that the infertility of the sex-reversed rabbits was due to the abnormal development of the ovaries. This is consistent with previous studies, suggesting that infertility is a common symptom of male-to-female sex reversal syndrome [4].

### Hormone assay of *SRY*-Sp1 KO, XY rabbits

It is well known that sex hormones play a crucial role in follicle formation, embryo implantation and pregnancy. To examine whether the KO, XY rabbits had endocrine features characteristic of females, we tested their sex hormone levels using ELISA. Endocrine data showed that testosterone (TSTO) levels in the KO, XY rabbits were significantly reduced compared to the WT, XY rabbits and in the range of the WT, XX controls (Figure 3B). Lower estradiol (E2), which can stimulate the development and maintenance of female reproductive tissues, was detected in the KO, XY rabbits (Figure 3C). Alternatively, higher levels of follicle-stimulating hormone (FSH), a hormone that regulates development, growth, pubertal maturation, and reproductive processes of the body, were observed in KO, XY rabbits (Figure 3D). Moreover, reduced prolactin (PRL) and progesterone (P) were detected in sex-reversed KO, XY rabbits (Figure 3E-3G). These results suggest that developmental disorders of the ovary may be responsible for aberrant levels of sex hormones, leading to infertility in KO, XY rabbits.

### The sex-reversed rabbit was successfully impregnated using embryo transfer and gave birth

Since no significant differences were observed in the oviduct, uterus or cervix between sex-reversed KO, XY and WT, XX rabbits, we suspected that the KO, XY rabbits could become pregnant though embryo transfer.

**Figure 4:**
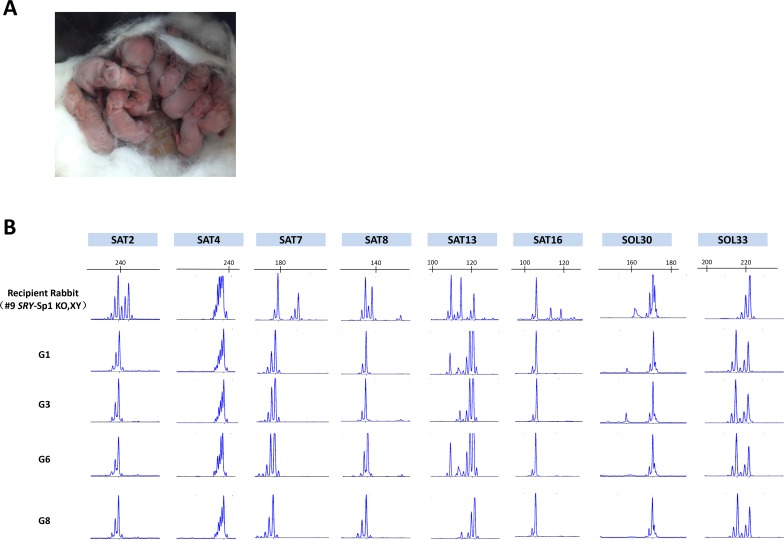
Generation of F1 s using a sex-reversed rabbit as a surrogate **A**. Picture of the F1 s from a sex-reversed rabbit. **B**. Representative microsatellite loci were analyzed in the recipient (#9 KO rabbit), the donor female rabbit and 4 of the pups from recipient rabbit #9 (G1, G3, G6 and G8).

To test this possibility, 135 high quality embryos from Rex rabbits were transferred to the oviduct of three pseudo-pregnant KO, XY rabbits. One of these recipients (#9) carried the pregnancy to term and gave birth to 12 live pups (Figure 4A and Table 2). No significant differences were observed in the birth weight or growth between the F1 progeny and normal rabbits. Microsatellite analysis confirmed that the genotypes of the newborn rabbits were distinct from the KO, XY rabbit (Figure 4B), suggesting that sex reversal syndrome patients might be able to have successful pregnancies using embryo transfer in a clinical setting.

**Table 2:** Summary of embryos transfer using the *SRY*-sp1-KO rabbits as recipient

Recipients	Species	Mutated sp1 binding site	Phenotype	Species of donor zygote	Embryos transferred	Pregnancy	Pups obtained
#9	NZW rabbit	Sp1-A,Sp1-C	Sex reversal	Beaver rabbit	45	Yes	12
#13	NZW rabbit	Sp1-A, Sp1-B, Sp1-C	Sex reversal	Beaver rabbit	48	No	
#18	NZW rabbit	Sp1-A, Sp1-B	Sex reversal	Beaver rabbit	42	No	

NZW rabbit: New Zealand white Rabbit

## DISCUSSION

Consensus sequences for Sp1 have been identified in the 5′ flanking regions of a variety of genes, although their roles in gene regulation are not yet well understood [7]. Sp1 is a major transcription factor of housekeeping genes, with the Sp1 consensus sequence commonly found flanking the 5′ regions of these genes [11]. In this study, three consensus Sp1 binding sequences were identified close to the transcription initiation site (TSS) in the 5′ region of the *SRY* gene. We discovered that two of these sites (Sp1-B and Sp1-C) may contribute to the expression of *SRY* gene, a finding consistent with a previous study [12]. Here, we demonstrated that mutation of the Sp1 binding site upstream of *SRY* induces sex reversal in rabbits, strengthening the association between Sp1 binding site mutations and sex reversal in the rabbit model.

Several recent studies have reported that there are at least two copies of *SRY* in the rabbit genome, which are located on the arms of an inverted repeat [13]. In this study, sgRNAs were designed to target the two *SRY* loci, and through comparative sequencing, the Sp1 binding sites of both *SRY* genes were deleted in the founder rabbit using the CRISPR/Cas9 system. This mutant successfully displayed the sex reversal phenotype, demonstrating that the CRISPR/Cas9 system is an excellent tool to direct fragment deletions in paralogous palindromic sequences. More studies are needed to determine whether both *SRY* copies are required for male sex development or if one copy is sufficient.

The same external and internal genitalia, copulatory behaviors and sex hormones were observed in the KO, XY rabbits compared to the WT, XX rabbits. The mechanisms behind the smaller ovaries, reduced follicles and infertility in KO, XY rabbits have not been investigated. Previous studies have reported that *SRY* KO mice were sterile or had reduced fertility, even though they had oocytes in their ovaries and performed copulatory behaviors as normal females [14, 15]. Therefore, we concluded that abnormal development of the ovaries and a reduced number of ovulated oocytes were responsible for the infertility of the sex-reversed rabbits. Furthermore, significantly decreased estrogen and aberrant sex hormone expression in the *SRY*-Sp1 KO, XY rabbits indicated that sexual maturation in sex reversal syndrome patients might be ovulation related.

Taken together, our observations provide the first evidence that deletion of two Sp1-binding sites located in the 5′ region of *SRY* induces sex reversal in rabbits and that a reduced number of ovulated oocytes is responsible for the infertility of sex-reversed rabbits. This novel animal phenotype can serve as an appropriate model for clinical research of male-to-female sex reversal syndrome. Furthermore, our results suggest that sex-reversed females could be successful impregnated and give birth using embryo transfer, upon consent.

## MATERIALS AND METHODS

### Ethics statement

New Zealand white rabbits and Rex rabbits were obtained from the Laboratory Animal Center of Jilin University (Changchun, China). All animal studies were conducted according to experimental practices and standards approved by the Animal Welfare and Research Ethics Committee at Jilin University.

### Cell culture and DNA transfection

Rabbit fetal fibroblast cells (RFF) were cultured in Dulbecco's Modified Eagle's Medium (DMEM) and incubated at 37°C in an atmosphere of 5% CO2. The cells were transfected using TurboFectTM *in vitro* Transfection Reagent (Fermentas) according to the manufacturer's instructions. The transfected cells were cultured in selective medium using 2.0 mg/ml puromycin (Sigma-Aldrich), and individual colonies were detected by sequencing the PCR products covering the target locus.

### Vector construction and *in vitro* transcription

Two complementary DNA oligonucleotides were annealed at 95°C for 5 min to generate double-strand DNA then cloned into a BbsI-digested pUC57-simple vector expressing Cas9 (Addgene ID 51307 for cells and Addgene ID 51307 for zygotes). In vitro transcription was performed as described previously [16].

### Embryo collection, microinjection, and transfer

The protocol for microinjection of pronuclear-stage embryos has been described in detail in our published protocols [16]. Briefly, a mixture of Cas9 mRNA (200 ng/ul) and sgRNA (50 ng/ul) was co-injected into the cytoplasm of pronuclear-stage zygotes. These zygotes were then immediately transferred into the oviducts of surrogate rabbits.

### Mutation and off-target effect detection assay

The protocol for the mutation and off-target effects detection assay has been described previously [16]. PCR primers are listed in Supplementary Tables S1 and S3. PCR products were gel purified and cloned into pGEM-T vectors (Tiangen, China). Ten positive plasmid clones were sequenced, and DNAman was used for sequence analysis.

### Quantitative real-time RT-PCR (qRT-PCR)

The protocol for RNA extraction has been described previously. *SRY* gene expression is presented as the mean ±SEM, as analyzed by 2^−ΔΔCT^ formula and GraphPad Prism software (T test). A probability of *p* < 0.05 was considered statistically significant, which was normalized to the amount of GAPDH mRNA. The primers used for qRT-PCR are shown in Supplementary Table S1.

### Histology

Hematoxylin and eosin (HE) staining were performed as previously described [17]. Briefly, the tissues were fixed with 4% paraformaldehyde for 48 h, embedded in paraffin wax, slide sectioned and then stained with hematoxylin and eosin and analyzed by microscope (Nikon TS100).

### Sex hormone assay

Eight-month-old *SRY*-Sp1 KO, WT, XX and WT, XY rabbits were anesthetized, and serum was obtained by precipitation and centrifugation. Sex hormones, including TSTO, E2, FSH, LH, P and PRL, were measured using an ELISA Kit (IBL, Germany). At least 4 rabbits from each group were used in this study, and all experiments were repeated three times. The data are expressed as the mean ± SEM.

### Microsatellite analysis

Microsatellite analysis was performed on the newborn rabbits and recipient rabbits [18]. Eight microsatellite loci located on different rabbit chromosomes were first visualized with 3% agarose gel electrophoresis and further confirmed by capillary gel electrophoresis with fluorescently-labeled amplimers and laser scanning using an ABI 3700 Genetic Analyzer and GeneMapper 4.0 (Applied Biosystems, Foster City, CA, USA). All primers for the microsatellite loci are shown in Supplementary Table S4.

### Statistical analyses

All data are expressed as the mean ±SEM, from at least three individual determinations in all experiments. The data were analyzed with one-way ANOVA using GraphPad Prism software 6.0. A probability of *p* < 0.05 was considered statistically significant.

## SUPPLEMENTARY MATERIALS FIGURES AND TABLES



## Abbreviations

293T: Human kidney epithelial cell lines
KO: knockout
HE: hematoxylin-eosin
POT: potential off-target
*dpp*: days post-partum
*dpc*: days post coitum
WT,XY: wild-type male control
WT,XX: wild-type female control
KO,XY: the SRY-Sp1 knockout male rabbits
TSTO: testosterone
E2: estradiol
FSH: follicle-stimulating hormone
PRL: prolactin
P: progesterone
LH: luteinizing hormone
