# Supplementary material for: Mutation of the Sp1 binding site in the 5′ flanking region of SRY causes sex reversal in rabbits
Source: Oncotarget. 2017 Apr 9;8(24):38176–83. doi: 10.18632/oncotarget.16979 (PMC5503524; doi:10.18632/oncotarget.16979)
Supplement: Supplementary file 1 [file oncotarget-08-38176-s001.pdf]

Mutation of the Sp1 binding site in the 5′ flanking region of *SRY* causes sex reversal in rabbits

Supplementary Material

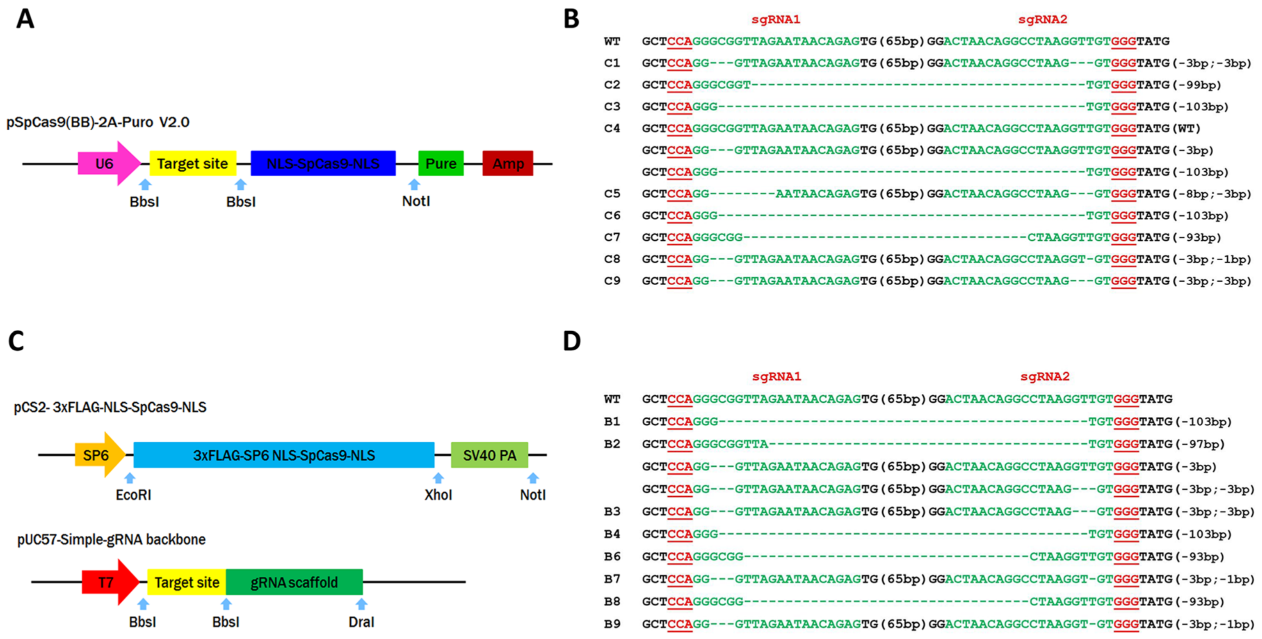

Supplementary Figure 1: CRISPR/Cas9-mediated mutation of Sp1 binding sites in cells and zygotes. (**A**) Constructs and schematic illustration of the CRISPR/Cas9 vector used for cell transfection. (**B**) T-cloning sequences of mutant Sp1 binding sites in cells. (**C**) Constructs and schematic illustration of the CRISPR/Cas9 vector used for zygote injection. (**D**) T-cloning sequences of mutant Sp1 binding sites in blastocyst. PAM sites are underlined and highlighted in red; target sequences are green; deletions (-) and insertions (+) are shown. WT, wild-type control.

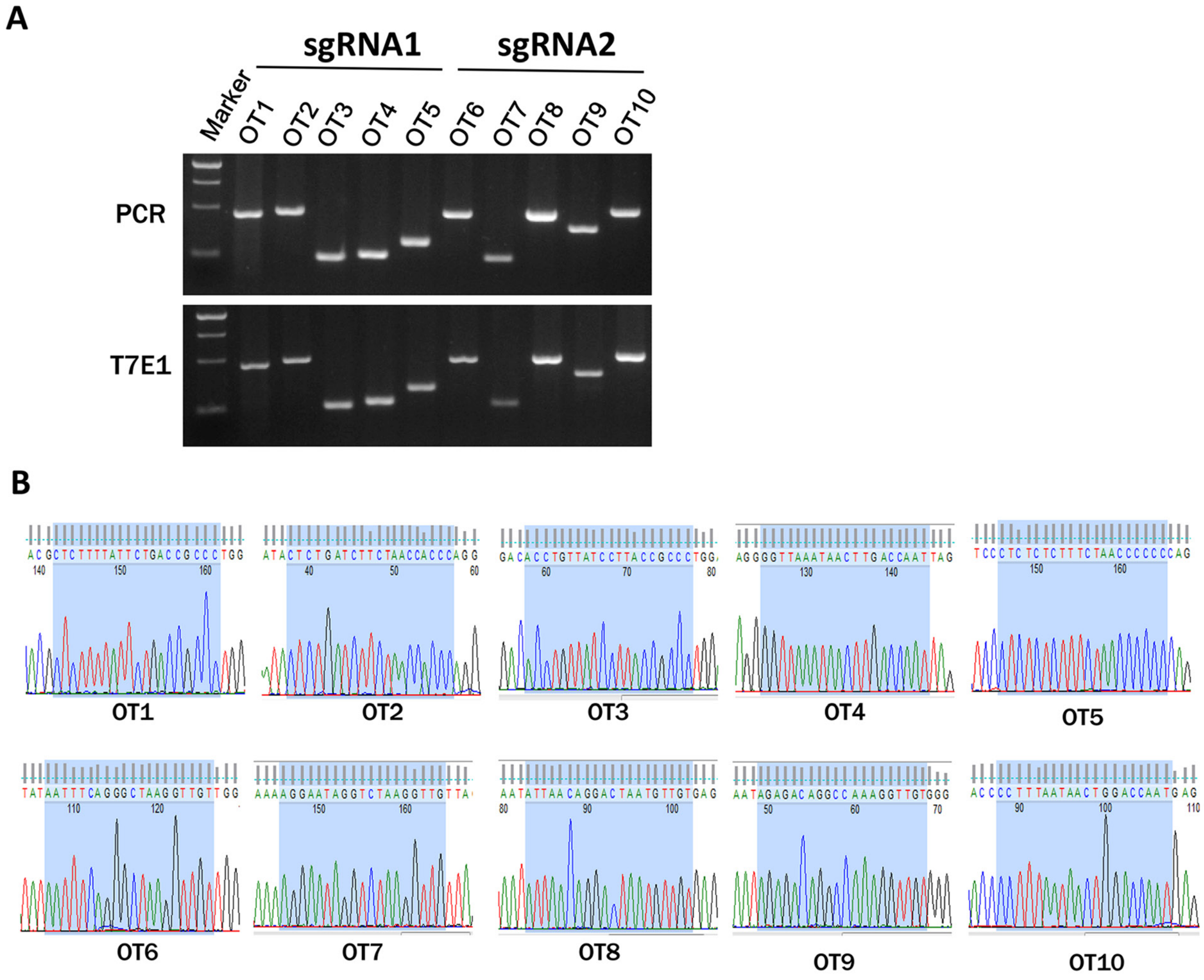

Supplementary Figure 2: Off-target analysis of the 2 sgRNAs in the SRY-Sp1KO rabbit. (**A**) T7E1 cleavage analysis of potential off-target sequences (POTS). (**B**) A chromatogram sequence analysis of POTS. The 20 bp of the POTS and the PAM are represented in shadow.

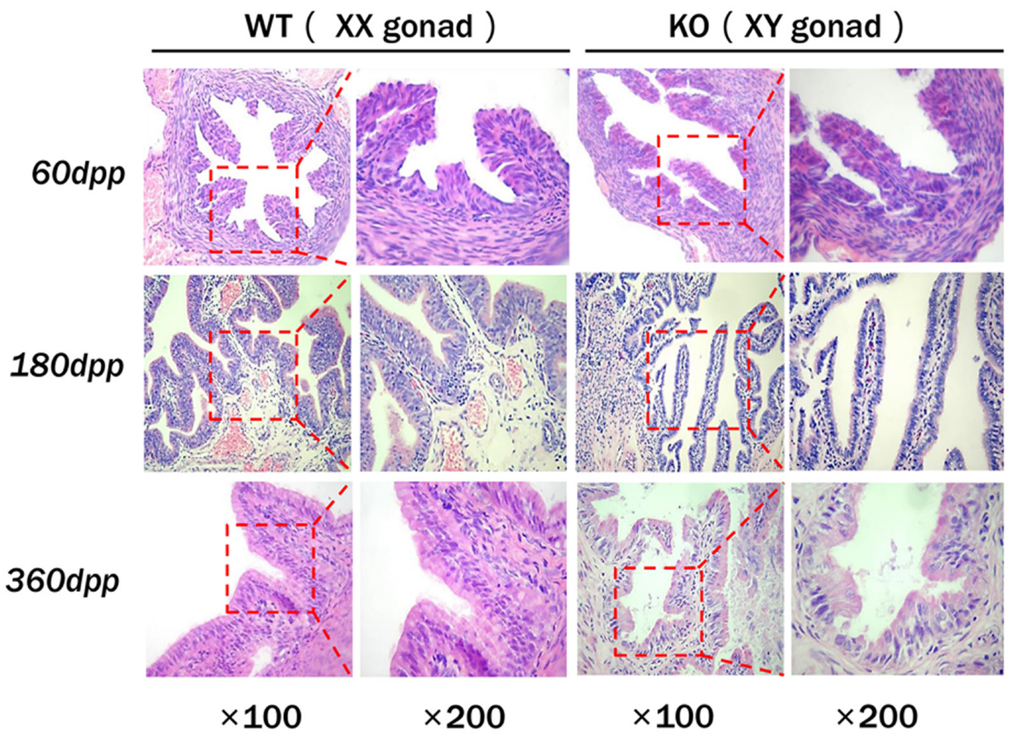

Supplementary Figure 3: H&E staining of the oviduct from the WT,XX and KO,XY rabbit at 60, 180 and 360dpp. No significantly difference were determined between WT and *SRY*-Sp1 KO,XY rabbit. WT,XX, wild-type female control; KO,XY, the *SRY*-Sp1 KO rabbits.

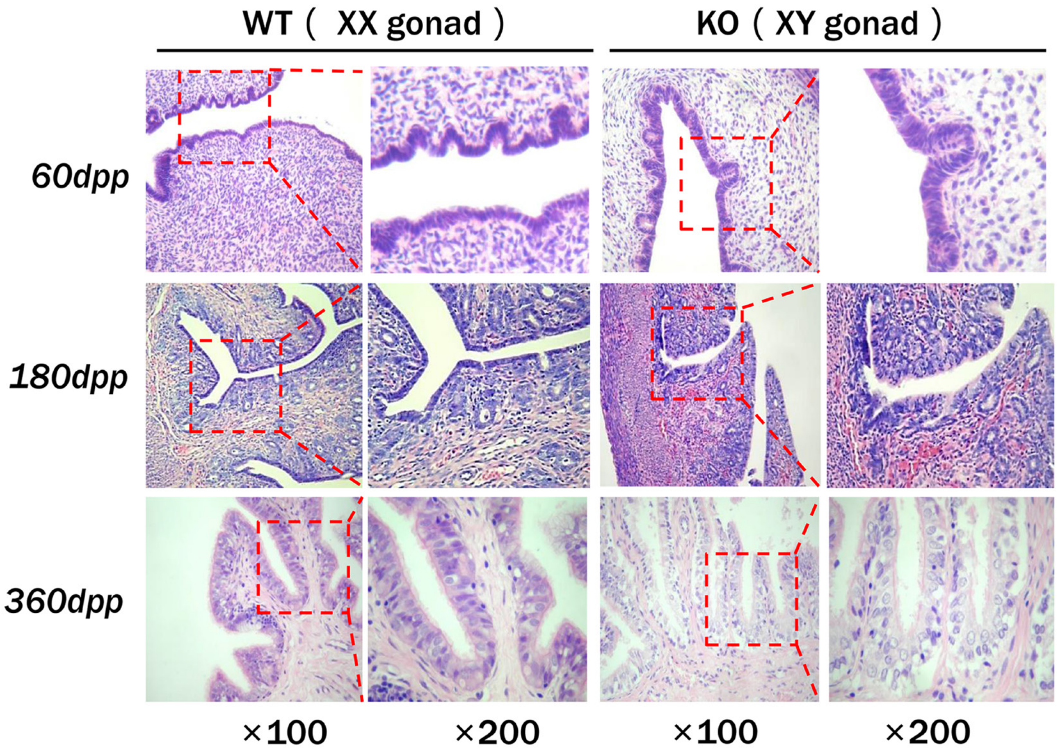

Supplementary Figure 4: H&E staining of the uterus from the WT,XX and KO,XY rabbit at 60, 180 and 360dpp. No significantly difference were determined between WT and *SRY*-Sp1 KO,XY rabbit. WT,XX, wild-type female control; KO,XY, the *SRY*-Sp1 KO rabbits.

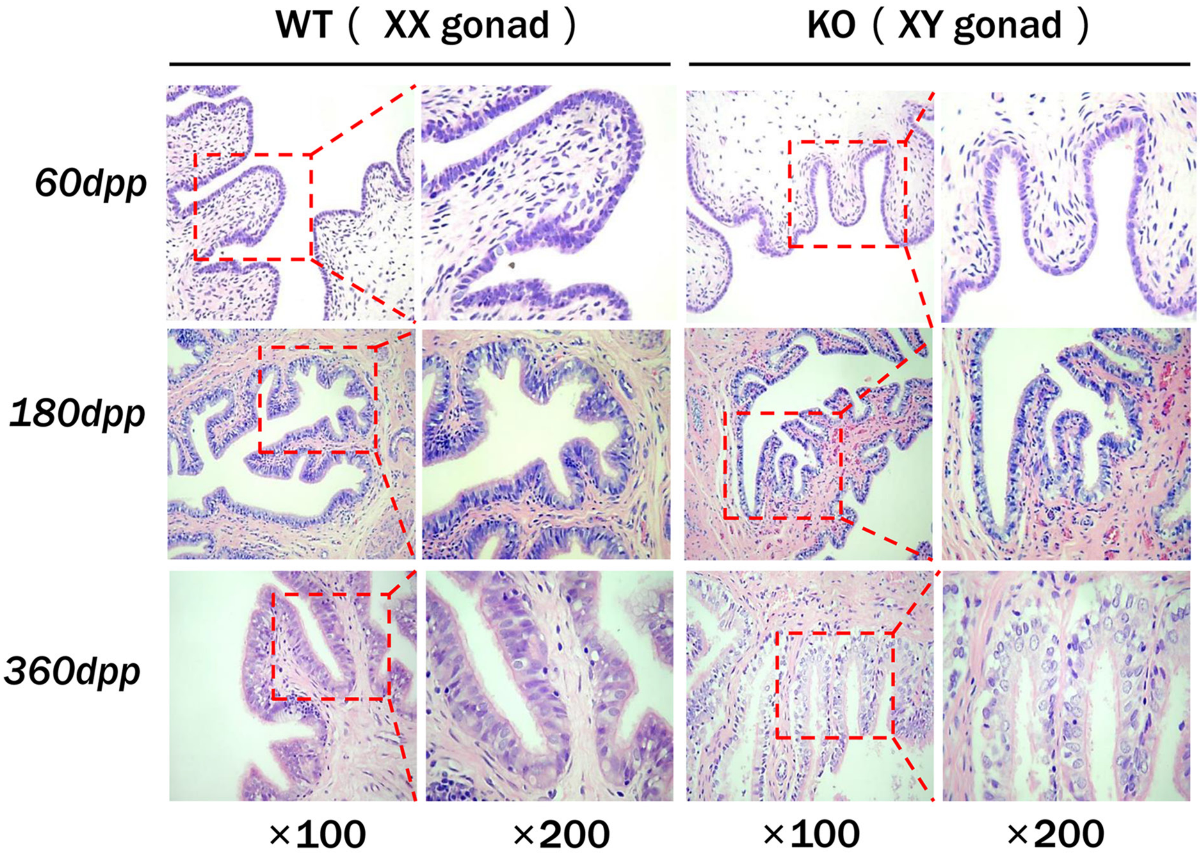

Supplementary Figure 5: H&E staining of the cervixs from the WT,XX and KO,XY rabbit at 60, 180 and 360dpp. No significantly difference were determined between WT and *SRY*-Sp1 KO,XY rabbit. WT,XX, wild-type female control; KO,XY, the *SRY*-Sp1 KO rabbits.

For Supplementray Tables 1,2,3,4 see in Supplementary Files
